# Detecting adenosine triphosphate in the pericellular space

**DOI:** 10.1098/rsfs.2012.0101

**Published:** 2013-06-06

**Authors:** Simonetta Falzoni, Giovanna Donvito, Francesco Di Virgilio

**Affiliations:** Department of Morphology, Surgery and Experimental Medicine, Section of General Pathology, University of Ferrara, Via Borsari 46, Ferrara 44121, Italy

**Keywords:** luciferase, bioluminescence, extracellular adenosine triphosphate, cancer, inflammation

## Abstract

Release of adenosine triphosphate (ATP) into the extracellular space occurs in response to a multiplicity of physiological and pathological stimuli in virtually all cells and tissues. A role for extracellular ATP has been identified in processes as different as neurotransmission, endocrine and exocrine secretion, smooth muscle contraction, bone metabolism, cell proliferation, immunity and inflammation. However, ATP measurement in the extracellular space has proved a daunting task until recently. To tackle this challenge, some years ago, we designed and engineered a novel luciferase probe targeted to and expressed on the outer aspect of the plasma membrane. This novel probe was constructed by appending to firefly luciferase the N-terminal leader sequence and the C-terminal glycophosphatidylinositol anchor of the folate receptor. This chimeric protein, named plasma membrane luciferase, is targeted and localized to the outer side of the plasma membrane. With this probe, we have generated stably transfected HEK293 cell clones that act as an *in vitro* and *in vivo* sensor of the extracellular ATP concentration in several disease conditions, such as experimentally induced tumours and inflammation.

## 1. Introduction

For many years, adenosine triphosphate (ATP) was solely considered for its role as the main source of energy in living cells; however, we now know that ATP also plays a fundamental physiological role as a pleiotropic extracellular messenger of cell-to-cell communication acting at plasma membrane receptors named P2 purinergic receptors [1]. The purinergic receptor family is composed of adenosine (P1) and nucleotide (P2) selective receptors. P1 receptors are further subdivided into A1, A2a, A2b and A3, whereas P2 receptors are subdivided into the P2Y and P2X subfamilies [2–4].

The P2Y subfamily has eight members: P2Y1, P2Y2, P2Y4, P2Y6, P2Y11, P2Y12, P2Y13 and P2Y14. The P2Y1 receptor is activated by adenosine diphosphate (ADP), whereas, at P2Y2, ATP and uridine-5′-triphosphate (UTP) are equipotent. At P2Y4 and P2Y6, the uridine nucleotides UTP and uridine diphosphate (UDP) are preferred agonists, respectively. P2Y12 and P2Y13 are selectively activated by ADP, and P2Y14 is activated by UDP–glucose or UDP–galactose [5]. The P2X subfamily includes seven receptors, P2X1–P2X7, for which ATP is the primary endogenous ligand [6]. Affinity for extracellular nucleotides ranges from the low nanomolar level (P2Y receptors) to the high micromolar level (P2X7 receptor). This wide range of affinities of P2 receptors for extracellular nucleotides confers a remarkable plasticity to purinergic signalling, allowing the detection of minute as well as large changes of agonist concentration within the extracellular space. Furthermore, ubiquitous distribution in all tissues makes P2 receptors one of the most common and versatile signalling systems in the human body. Thus, it is not surprising that extracellular ATP, ADP and UTP are involved in a wide variety of different responses such as cell proliferation, migration and differentiation, neurotransmitter and cytokine release, necrosis and apoptosis [7]. Likewise, nucleotide signalling participates in several crucial physiological and pathological events such as embryonic development, immune system maturation, neurodegeneration, inflammation and cancer [8].

Their intrinsic features make nucleotides the ideal signal molecules to report cell damage or distress (damage-associated molecular patterns; DAMPs) as they are concentrated to high levels within the cell cytoplasm, but virtually absent in the extracellular space [9]. In addition, to further support their role as DAMPs, nucleotides, being charged species, are highly diffusible through the aqueous interstitial tissue and quickly hydrolysed by specialized degrading systems, as expected of any bona fide biological messenger. Last but not least, nucleotides ligate specific plasma membrane receptors that confer a remarkable specificity to their signalling.

The nucleotide-degrading system plays a critical role in purinergic signalling because, besides degrading ATP, and therefore terminating P2 receptor-targeted signalling, it also generates adenosine, an additional powerful modulator of cell functions acting at P1 receptors [10]. The main enzymes involved in ATP hydrolysis and adenosine generation are the ubiquitous ecto-nucleotidases CD39, which converts ATP and ADP to adenosine monophosphate (AMP), and CD73, which converts AMP to adenosine. Thus, in principle, the extracellular ATP concentration can change as a consequence of enhanced ATP release as well as of reduced ATP hydrolysis. ATP de novo synthesis owing to adenylate kinase, nucleoside diphosphate kinase and ATP synthase expressed on the outer aspect of the plasma membrane might also contribute to the accumulation of extracellular ATP, but these latter pathways are as yet poorly characterized [11].

Although the intracellular ATP concentration is in the millimolar range (3–10 mM), the extracellular concentration is considerably lower. It is estimated that the physiological ATP concentration in human blood is normally submicromolar (20–100 nM) [12,13], although it is reported to increase after sustained exercise [13]. Measurement of extracellular ATP within tissue interstitium is much more technically demanding and uncertain; however, it is reckoned that quiescent cells keep the pericellular ATP concentration in the low nanomolar range. It is worth stressing that this is likely to be an imprecise estimate. In fact, increasing evidence suggests that cells are surrounded by a halo of ATP, with a higher concentration within the unstirred layer closer to the cell surface [14,15]. Accurate measurement of ATP levels within this layer, which contains the actual ATP concentration ‘seen’ by plasma membrane P2 receptors, is extremely difficult. Indirect experimental evidence suggests that in the pericellular halo the ATP concentration is sufficient to keep most P2 receptors in a state of tonic activation, the low affinity P2X7 included, to the point that some of these receptors might even be partially desensitized [16]. The extracellular ATP concentration can increase in response to any cell perturbation owing to physical, chemical or biological stimuli, to reach the hundred micromolar level in many disease states such as ischaemia, hypoxia, trauma, cancer or inflammation [17].

Pathways for ATP release are diverse, though poorly characterized. There is no doubt that, thanks to its favourable chemical concentration gradient, ATP can easily passively efflux out of the cell. Thus, a number of candidate ATP-permeable release channels have been put forward, e.g. VDAC [18] or other chloride channels such as the cystic fibrosis transmembrane conductance channel regulator [19], ABC transporters [20], connexins [21,22], pannexins [23] and the P2X7 receptor itself [24]. Moreover, ATP can also be actively released via vesicular release from mast cells, platelets, neurons and in theory from any cell capable of stimulated or constitutive exocytosis [22]. Of course, large amounts of ATP may be released from injured or necrotic cells [25]. Recently, it has been shown that autophagy-competent cells also release ATP, which might be a constituent of the peculiar biochemical microenvironment of tissues undergoing autophagy [26]. In general, it is worth stressing that ATP release also occurs in response to a variety of even minor mechanical stresses owing to routine experimental procedures (cell rinsing, medium changing). Despite the generally acknowledged important role of extracellular ATP, only a few tools are available today for its quantification in physiological or pathological conditions.

Usually, extracellular ATP is measured in the cell supernatant by using the standard bioluminescence luciferine/luciferase assay. However, this technique gives only an indirect estimate of the level that ATP can reach at sites of release close to the plasma membrane, and, more importantly, does not allow real-time or *in vivo* measurement of the extracellular ATP concentration. Thus, there is a need to develop novel probes/techniques that allow closer monitoring of ATP kinetics in the extracellular space. The pioneering technique of Dale and co-workers [27] is microelectrode recording. This approach is simple, accurate, quantitative and amenable to *in vivo* measurements, but has a major drawback: sticking an electrode into a tissue unavoidably causes a certain amount of damage that affects the ATP measurement. Dubyak and co-workers [14] proposed a method for real-time measurement of ATP by using a cell-surface-bound luciferase. Firefly luciferase was fused in frame with the immunoglobulin G (IgG) binding domain of *Staphylococcus aureus* protein A (a construct named proA-luc), thus allowing this chimeric protein to bind to IgG adsorbed on the surface of cells via interaction with native antigens. The feasibility of proA-luc as a cell surface ATP-measuring probe was validated in three cell systems: human platelets, HL-60 promyelocytic cells and Bac-1.2F5 macrophages. An improvement of this technique has been described by Kobatake and co-workers [28]. A more sophisticated approach was proposed at about the same time by Schneider and co-workers [29]. These authors engineered a scanning tip coated with the ATPase-containing S1 myosin fragment and exploited atomic force microscopy to identify point sources of ATP release at the surface of living cells and to measure the local ATP concentration. This rather complex measuring technique might have been difficult to apply, as, to the best of our knowledge, it has not been used in subsequent studies. Another biosensor method was developed by Hayashi and co-workers [15]. The method is based on the measurement of ATP-dependent currents of P2X2 channels expressed on a sensor cell, patched on a patch-clamp micropipette and placed near the ATP-releasing target cell. Based on P2X2 receptor affinity for ATP, this technique allows a fairly accurate quantification of the extracellular ATP concentration. A calibration curve may be constructed by local application of known ATP concentrations. Other methods use fluorescence microscopy for real-time ATP measurement by a two-enzyme system. Corriden *et al.* [30] reported a technique based on a tandem enzyme reaction driven by hexokinase and glucose-6-phosphate dehydrogenase, which, in the presence of ATP and glucose, converts nicotinamide adenine dinucleotide phosphate (NADP) to NADPH. This latter nucleotide, being fluorescent, can be imaged by fluorescence microscopy. Rather interestingly, with this method, the authors were able to show that ATP may reach concentrations of up to 80 μM in the vicinity of the plasma membrane.

## 2. Bioluminescence

**Figure 1.**
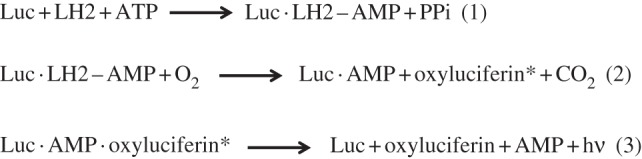
Reaction steps leading to ATP-driven luciferase (LUC)/luciferin (LH2) photoemission.

Luciferase reporters as a source of bioluminescence are by far the most widely used probes for the measurement of ATP, whether in free solution, within intact isolated cells or *in vivo* [31]. Bioluminescence is a natural phenomenon owing to chemical emission of light (chemiluminescence), remarkably conserved across a variety of different species (bacteria, protists, fungi, insects, a variety of marine organisms) with the notable exception of higher terrestrial organisms. Chemically, this process yields photons as a consequence of an exergonic reaction catalysed by a class of enzymes (e.g. luciferases) that oxidize a photon-emitting substrate (luciferin). In nature, there are different types of light-emitting enzymes (e.g. luciferases, aequorin), each with a specific substrate selectivity. In the course of time, many luciferases have been isolated and characterized from several sources and, to date, luciferase reporter systems are extensively used *in vitro* and *in vivo* to investigate gene expression [32], track cancer cells in living animals [33] or measure environmental pollutants [34]. The most widely used luciferases are firefly luciferase (Fluc) from *Photinus pyralis* and *Renilla* luciferase (Rluc) from *Renilla reniformis*. Firefly luciferase is a 62 kDa protein member of the adenylating enzyme superfamily. In the presence of its substrate d-luciferin (LH2), Mg^2+^ ion, molecular oxygen and ATP, this enzyme catalyses a multi-step reaction that yields light in the green-to-yellow region (**λ**_max_ = 560 nm) [35]. The first step involves the initial formation of the intermediate enzyme–d-luciferyl adenylate (d-LH2–AMP), with release of inorganic pyrophosphate. Subsequently, this intermediate is oxidized by molecular oxygen with the formation of carbon dioxide and the excited complex enzyme–oxyluciferin–AMP. In the last step of the reaction, the rapid loss of energy from the excited complex produces photon emission with dissociation of the individual components (figure 1). For each quantum of light emitted 1 mol of ATP, 1 mol of oxygen and 1 mol of luciferin are consumed.

For its high sensitivity and specificity, firefly luciferase has found numerous applications in biomedicine, but it is above all the most important sensor of cellular ATP. As such, luciferase is widely used in whole cell lysates to measure the intracellular ATP concentration, and, more recently, it has also been used to monitor ATP release into cell supernatants. Measurement of the extracellular ATP concentration has rapidly become a frequent application of luciferase, given the increasing importance that purinergic signalling has recently achieved in cell biology [8,36].

Measuring ATP release in cell supernatants with soluble luciferase has two major limitations: in the first place, sample manipulation causes a perturbation that by itself might cause an unwanted cell stimulation with consequent release of ATP; second, soluble luciferase is likely to be unable to detect rapid changes in the concentration of ATP close to the plasma membrane, i.e. exactly where extracellular ATP is biologically active. In order to overcome these technical drawbacks and be able to investigate the dynamic changes of extracellular ATP *in vitro* and *in vivo*, in 2005, we engineered a chimeric luciferase targeted to and retained on the external side of the plasma membrane named plasma membrane extracellular luciferase (pmeLUC) [24].

## 3. The pmeLUC probe

**Figure 2.**
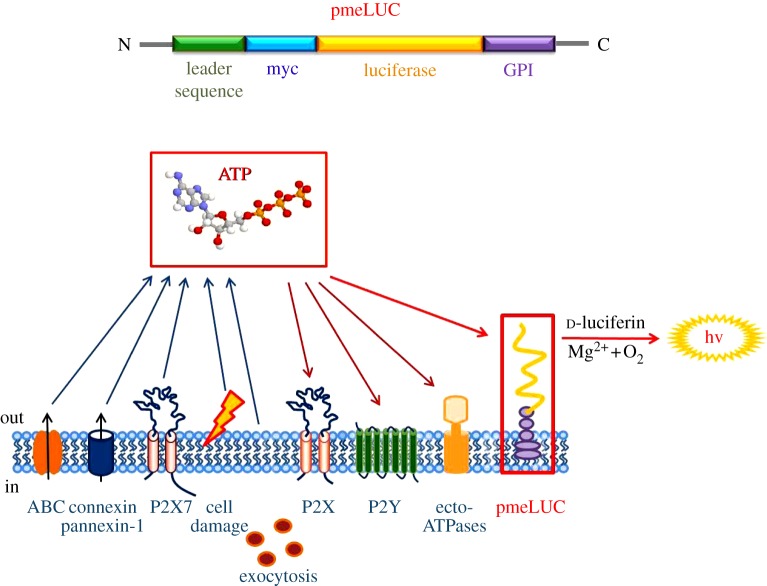
Membrane topology of pmeLUC. The pmeLUC construct is composed of the full-length coding sequence of luciferase (yellow) inserted in-frame between the N-terminal leader sequence (green) and the C-terminal GPI anchor (violet) of the folate receptor. A c-myc tag (light blue) is also added in-frame for tracking purposes. The pmeLUC protein is targeted and localized to the outer side of the plasma membrane, in close vicinity to all the molecules participating in purinergic signalling (adapted from Pellegatti *et al*. [24]).

The pmeLUC probe was devised as an important analytical tool to measure extracellular ATP in the vicinity of the cell surface. It is a chimeric protein in which the luciferase cDNA (from *Photinus pyralis*) was fused in frame between the N-terminal sequence encoding an endoplasmic reticulum-targeting signal (26 amino acid (aa) long leader sequence) and the C-terminal plasma membrane anchor sequence (glycophosphatidylinositol (GPI); 28 aa long anchor sequence), both derived from the folate receptor. Thanks to these modifications, the pmeLUC probe is targeted and expressed on the plasma membrane, with the catalytic site facing the extracellular milieu. This topology enables pmeLUC to measure ATP increases owing to transient release in the close vicinity of the plasma membrane [24] (figure 2).

**Figure 3.**
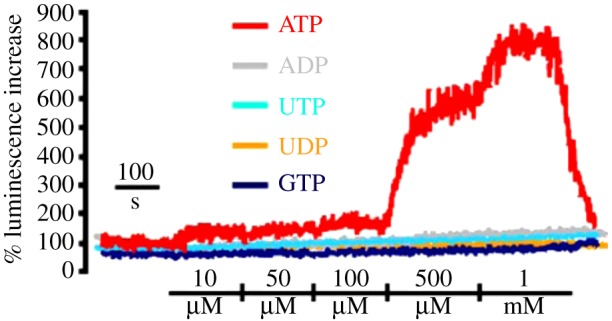
Nucleotide selectivity of plasma membrane-expressed luciferase (pmeLUC). HEK293-pmeLUC cells were placed in a luminometer chamber and perfused with solutions containing increasing nucleotide concentrations as described in Pellegatti *et al*. [24] (adapted from Pellegatti *et al*. [24]).

**Figure 4.**
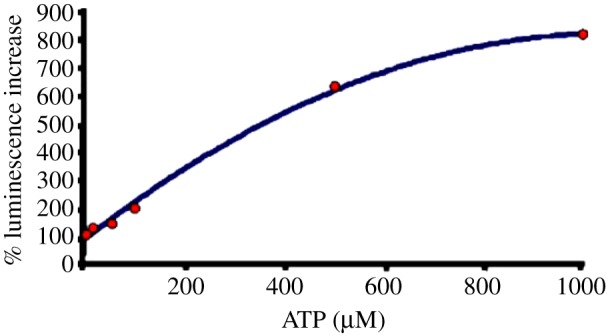
ATP calibration curve of plasma membrane-expressed luciferase (pmeLUC). HEK293-pmeLUC cells were placed in a luminometer chamber and perfused with solutions containing increasing ATP concentrations as described in Pellegatti *et al*. [24] (adapted from Pellegatti *et al*. [24]).

pmeLUC can be transfected into a variety of cell types to generate stable clones (e.g. HEK293-pmeLUC or CT26-pmeLUC cells) for *in vitro* and *in vivo* experiments. HEK293-pmeLUC cells were among the first stable clones that we generated, and in which the pmeLUC probe was extensively validated. We first carried out an extensive *in vitro* characterization which showed that this probe is insensitive to other nucleotides, such as ADP, UTP, UDP and guanosine triphosphate (GTP) (figure 3), and ATP selective. Rather surprisingly, affinity is low compared with soluble luciferase, as the ATP threshold is in the low micromolar range (5–10 μM), with saturation of the signal at near millimolar ATP levels. An ATP calibration curve can be obtained that provides a useful reference for *in vitro* as well as *in vivo* experiments (figure 4). The pmeLUC probe efficiently measures ATP release triggered by pharmacological or mechanical stimuli. Last but not least, pmeLUC-transfected cells proliferate normally *in vitro* and respond to a variety of physiological agonists tested [24]. An important advantage of this ATP sensor is its feasibility for *in vivo* imaging of extracellular ATP. In fact, pmeLUC-transfected cells can be inoculated into test animals and used as *in vivo* probes of the ATP concentration within tissue interstitium. Of course, as usual for all bioluminescence applications, luciferine (usually 3 mg per mouse) must also be routinely injected intraperitoneally (i.p.) before luminescence acquisition. This standard procedure is absolutely harmless and very reliable because luciferine is non-toxic and freely diffusible through the tissues. As detailed in §4, we have used HEK293-pmeLUC to image ATP levels at tumour and inflammation sites in living animals with a total body luminometer. An obvious drawback of using pmeLUC-transfected cells as an *in vivo* probe is the unavoidable stimulation of the immune response against the allogeneic cells (HEK293 are human cells). To circumvent this problem, in the first *in vivo* application, HEK293-pmeLUC cells were injected into *nude/nude* mice [37], in order to reduce the immune reaction. In the *nude/nude* host, HEK293-pmeLUC cells remain viable and functional for over a month [37]. This drawback can be partially circumvented by generating pmeLUC-transfected cell clones syngeneic with the host (see below), but yet luciferase will be identified as an alloantigen by the immune system. However, pmeLUC-transfected cancer cells produce tumours in the syngeneic host with kinetics comparable to that of control, pmeLUC-negative cancer cells [26], but no extensive studies have as yet been performed to monitor the long-term fate of injected pmeLUC cells in an immunocompetent host. The pmeLUC probe allows real-time measurement of the biochemical composition of inflammatory and tumour microenvironments, and imaging of extracellular ATP changes occurring over an extended length of time. As for HEK293, different cell types can be engineered to express pmeLUC; for example, transfecting inflammatory or cancer cells with pmeLUC might allow the inflammatory or tumour microenvironment to be probed directly *in vivo*, respectively. Alternatively, pmeLUC-transfected cells can be directly inoculated into the tissue site of interest to report the local extracellular ATP concentration. Obviously, a limitation in the use of the pmeLUC probe is the need for transfection, a technical step that narrows the cell types and processes amenable to investigation.

## 4. Applications

We first used pmeLUC-transfected cells to probe the ATP content of the tumour microenvironment [37]. Investigation of the biochemical composition of the tumour microenvironment is a focus of current interest as it is now clear that tumour progression and metastasis diffusion depend critically on the peculiar properties of this compartment. Here, a complex array of factors are secreted that inhibit cell death and promote survival and proliferation, stimulate angiogenesis, invasion and metastasis, and inhibit T-cell- and natural killer cell-mediated cytotoxic responses. Extracellular ATP is a key biochemical constituent of the tumour microenvironment. The tumour microenvironment is eminently hypoxic and it has long been known that hypoxia causes ATP release [38,39]. Furthermore, hypoxia-inducible factor-alpha has a strong modulatory effect on the expression of the extracellular enzymes that hydrolyse extracellular ATP and generate adenosine [40]. Thus, one of the hallmarks of the tumour microenvironment is its abundance of purinergic mediators. Very probably, ATP is released into the tumour microenvironment by inflammatory as well as tumour cells. Here, it is understood that this nucleotide may act as an autocrine/paracrine stimulus to support cell growth and differentiation [41–43].

**Figure 5.**
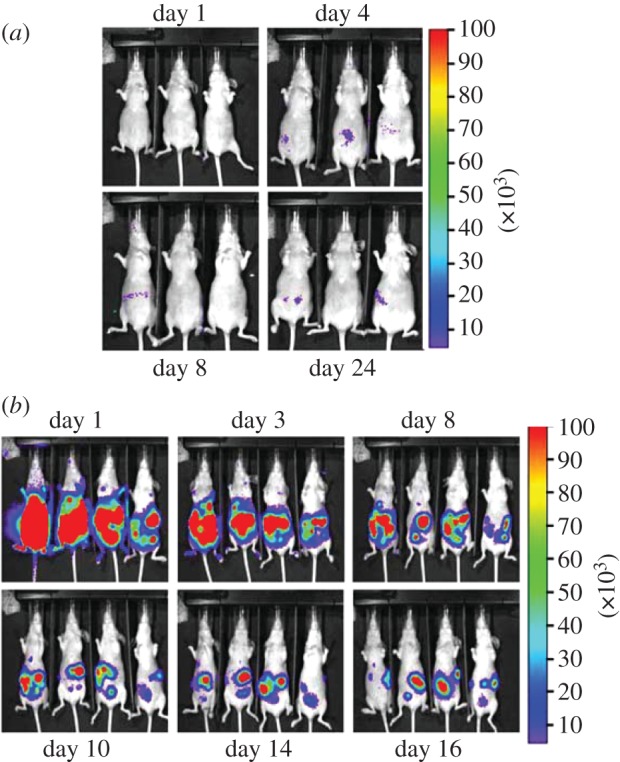
pmeLUC as a probe of ATP within the tumour microenvironment. Bioluminescence imaging of tumour-bearing nude mice injected with HEK293-pmeLUC cells. (*a*) Healthy nude mice were injected i.p. with 2 × 10^6^ HEK293-pmeLUC cells and monitored for 30 days. No luminescence was detected at any time. (*b*) *Nude/nude* mice were injected i.p. with the human ovarian carcinoma cell line OVCAR-3 (1.5 × 10^6^). Twenty days post-inoculum, HEK293-pmeLUC cells (2 × 10^6^) were injected i.p. and luminescence monitored for 16 days. As shown, strong luminescence emission was detected initially throughout the peritoneal cavity, and at later time points at discrete sites corresponding to tumour foci on the peritoneum (adapted from Pellegatti *et al*. [37]).

**Figure 6.**
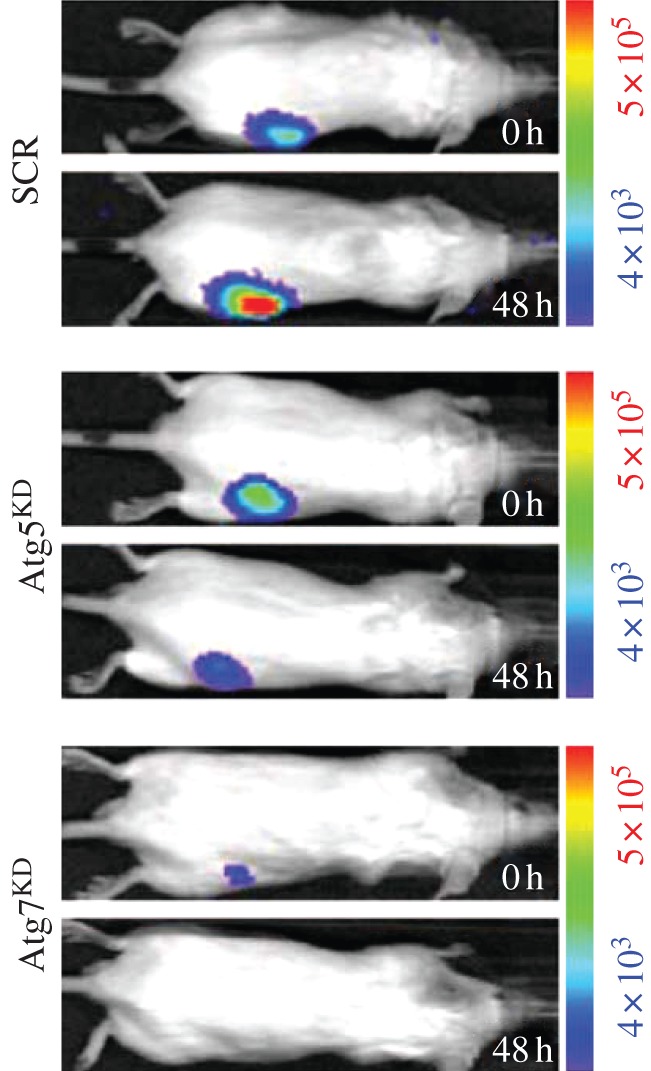
pmeLUC reveals an enhanced level of ATP release from autophagy-competent cancer cells. CT26 cancer cells engineered to express the pmeLUC exhibited a significant increase in ATP-dependent luminescence 48 h after systemic chemotherapy with the anti-cancer drug mitoxantrone. ATP release was much higher in autophagy-competent CT26 cells (SCR, cells transfected with a scrambled siRNA construct) than in autophagy-deficient CT26 cells transfected with a siRNA specific for the two autophagy genes Atg7 and Atg5 (adapted from Michaud *et al*. [26]).

ATP might be responsible for several responses that promote tumour progression: (i) induction of a distorted maturation of tumour-associated dendritic cells that would favour a T-helper 2 (Th2) rather than Th1 response; (ii) stimulation of tumour cell proliferation; (iii) potentiation of tumour cell aerobic glycolysis (Warburg effect); (iv) stimulation of release of angiogenic factors (e.g. vascular endothelial growth factor); and (v) generation of the potent immunosuppressor adenosine [44–46]. In the first place, we have used HEK293-pmeLUC cells to verify the assumption that tumour interstitium is ATP rich. To this aim, HEK293-pmeLUC cells were injected into tumour-bearing or control nude mice, and bioluminescence was monitored with a total body luminometer (Caliper-PerkinElmer IVIS Lumina) [37]. Very interestingly, injection of HEK293-pmeLUC cells into healthy animals produced no luminescence, irrespective of the route of inoculation, i.e. intravenous, intraperitoneal or subcutaneous (figure 5*a*). On the contrary, inoculation of the reporter cells into mice bearing the OVCAR-3 human ovarian carcinoma or the MZ2-MEL human melanoma produced strong luminescence at tumour sites (figure 5*b*). Luminescence was sensitive to injection of apyrase, a potent ATP-hydrolysing enzyme. Calibration of the luminescence signal revealed that the ATP concentration in the tumour interstitium reached the hundred micromolar range. Further demonstration of the reliability of pmeLUC cells as a sensor of extracellular ATP comes from experiments in which this probe was directly transfected into the CT26 colon carcinoma cells (CT26-pmeLUC cells), and these transfectants were used to establish the tumour [26] (figure 6). Also in this case, luminescence analysis revealed that tumour cells generate a microenvironment in which the ATP concentration is in the hundred micromolar range. Rather interestingly, these experiments revealed that ATP release from cancer cells correlates with other biological features, such as ability to carry out autophagy, that directly impinge on the tumour's susceptibility to chemotherapy. In addition, ATP release from cancer cells is also necessary to induce an efficient anti-cancer immune response via stimulation of the P2X7 receptor expressed by tumour-infiltrating dendritic cells or tumour-associated macrophages [47].

## 5. Inflammation

**Figure 7.**
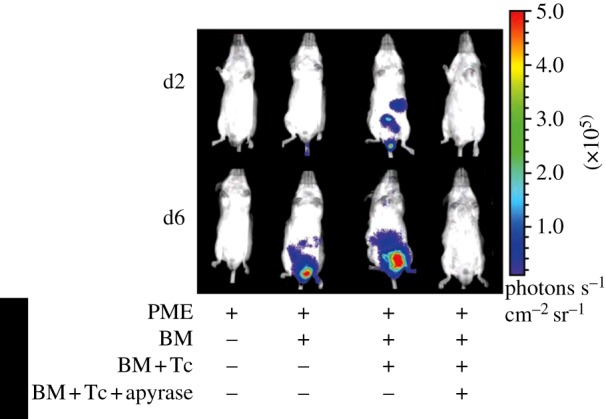
Increased ATP release in the gut during GVHD in mice. Extracellular ATP levels were revealed by HEK293-pmeLUC cells injected intravenously in BALB/c mice that were either untreated or had received irradiation and allogeneic bone marrow alone (BM), or BM together with allogeneic T cells (BM+Tc), or BM with allogeneic T cells and apyrase (BM+Tc+apyrase). These data indicate that the ATP level increases in the gut during GVHD. ATP increase is abrogated by apyrase (adapted from Wilhelm *et al*. [48]).

The tumour microenvironment has its own specific features, but there is no doubt that generally speaking it can be equated to an inflammatory milieu. Therefore, it is anticipated that inflammatory conditions of non-cancer origin are also characterized by a high extracellular ATP content. This was shown to be true in two models of inflammation: graft-versus-host disease (GVHD) and allergic contact dermatitis (ACD). Acute GVHD is a serious complication of allogeneic bone marrow transplantation and a major cause of morbidity and mortality. Understanding the mechanism that leads to excessive immune-mediated tissue destruction and to systemic inflammation is vital to design more effective therapeutic strategies. GVHD is often started by host tissue damage by transplant procedures, such as high-intensity chemoradiotherapy, that activate host antigen presenting cells (APCs), which are therefore already primed before donor tissue transplant. Total body irradiation is well known to stimulate host immune cells to secrete inflammatory cytokines such as tumour necrosis factor-α and interleukin-1β, and to induce endothelial and epithelial cell damage, especially in the gastrointestinal tract. In a second phase, host APCs present alloantigens to resting donor T cells, which are then activated and stimulated to proliferate. Together with T lymphocytes, macrophages and neutrophils are also activated in the target organs, thus further amplifying the inflammatory response and the subsequent tissue injury. Recent evidence shows that extracellular ATP plays a central role in the orchestration of this process [48]. Accordingly, increases in the extracellular ATP concentration can be demonstrated during GVHD, especially in the specific target organs (figure 7).

ACD is a T-cell-mediated inflammatory skin disease caused by low molecular chemicals or metal ions. The molecular mechanism by which contact allergens activate the innate immune response is largely unknown, but it is increasingly clear that activation of dendritic cells by locally released DAMPs has a key role in the generation of an efficient immunostimulation by allergens. In ACD, as in GVHD, Weber and co-workers [49] showed that an increase in the extracellular ATP concentration occurs before the early phases of the process, and that prevention of the ATP increase attenuates the inflammatory response [49].

## 6. Conclusion and future directions

Bioluminescence is an established and reliable technique for monitoring several biological parameters. The versatility of luciferase coupling to different physiologically relevant gene sequences and the availability of several genetically engineered mice that express luciferase either constitutively or conditionally allows visualization of a host of biological responses with little or no discomfort for the animal. The luciferase substrate, luciferin, is harmless, freely diffusible throughout the body and relatively cheap. pmeLUC was the obvious development of the luciferase/luciferin technique and of current efforts to better understand the pathophysiology of purinergic signalling. We believe that the ability to real-time visualize extracellular ATP *in vivo* should help solve several critical issues and allow a great leap forward in this field. The basic idea behind pmeLUC is indeed trivial, but establishing the optimal conditions for reliable and reproducible *in vitro* expression of functional pmeLUC required a substantial amount of experimental work. Nevertheless, we are now able to express functional pmeLUC in many human and mouse cell lines without major problems, except for the usual cell type limitations of cell transfection. On the contrary, and rather surprisingly to us, *in vivo* application of pmeLUC was much less problematic: the stable pmeLUC transfectants selected at the end of the cumbersome *in vitro* procedure described in Pellegatti *et al*. [24] turned out to be perfectly responsive and reliable the very first time they were tested *in vivo* [37]. Ease of use and reproducibility are certainly reasons for the increasing attention that the scientific community is paying to pmeLUC. As of February 2013, each of the two seminal papers describing the construction and *in vitro* validation of this probe [24] and its first *in vivo* application [37] received over 70 citations. Moreover, our group has freely provided both the pmeLUC plasmid and the stably transfected HEK293-pmeLUC cells to several laboratories that have independently validated this sensor *in vivo.* Results from some of these studies are already available in the literature [48–51]. The main advantage of pmeLUC is its cellular location that allows recording of ATP changes in a restricted milieu, close to the external surface of the plasma membrane, and not easily accessible to other probes. Low affinity for ATP, which in principle should be a drawback, turns out to be a bonus, because it makes pmeLUC responsive only to frankly pathological increases in extracellular ATP, thus making this probe a useful indicator of disease or injury (figure 5). Clearly, under certain conditions, pmeLUC-transfected cells might be unsuitable to reach the core of an inflammatory site or of a solid tumour. In this regard, a model mouse constitutively expressing pmeLUC would be ideal. In collaboration with the Danish Center for Genetically Modified Mice (Arhus, Denmark), we have generated a transgenic mouse that constitutively expresses pmeLUC. The pmeLUC mouse is currently being characterized in the authors' laboratory. A conditional pmeLUC mouse model would also further extend the range of applications. Furthermore, *in vivo* applications of this technique will certainly be facilitated by the availability of luciferases with increased thermostability [52], tolerance to acidic pH [53,54] or peak emission at longer wavelength [55,56], or novel more stable luciferin analogues [57].

In conclusion, we believe that the pmeLUC probe and its congeners will allow a more efficient exploration of protected environments that have been up to now refractory to biochemical investigation, will enhance our understanding of inflammation and tumour pathophysiology and will provide novel tools for imaging diseased sites *in vivo*.
